# Transcription factor programming of human ES cells generates functional neurons expressing both upper and deep layer cortical markers

**DOI:** 10.1371/journal.pone.0204688

**Published:** 2018-10-11

**Authors:** Giedre Miskinyte, Marita Grønning Hansen, Emanuela Monni, Matti Lam, Johan Bengzon, Olle Lindvall, Henrik Ahlenius, Zaal Kokaia

**Affiliations:** 1 Laboratory of Stem Cells and Restorative Neurology, University Hospital, Lund, Sweden; 2 Lund Stem Cell Center, Lund University, Lund, Sweden; 3 Stem Cells, Aging and Neurodegeneration Group, University Hospital, Lund, Sweden; 4 Division of Neurosurgery, Department of Clinical Sciences Lund, University Hospital, Lund, Sweden; University of Nebraska Medical Center, UNITED STATES

## Abstract

Human neurodegenerative disorders affect specific types of cortical neurons. Efficient protocols for the generation of such neurons for cell replacement, disease modeling and drug screening are highly warranted. Current methods for the production of cortical neurons from human embryonic stem (ES) cells are often time-consuming and inefficient, and the functional properties of the generated cells have been incompletely characterized. Here we have used transcription factor (TF) programming with the aim to induce rapid differentiation of human ES cells to layer-specific cortical neurons (hES-iNs). Three different combinations of TFs, NEUROGENIN 2 (NGN2) only, NGN2 plus Forebrain Embryonic Zinc Finger-Like Protein 2 (FEZF2), and NGN2 plus Special AT-Rich Sequence-Binding Protein 2 (SATB2), were delivered to human ES cells by lentiviral vectors. We observed only subtle differences between the TF combinations, which all gave rise to the formation of pyramidal-shaped cells, morphologically resembling adult human cortical neurons expressing cortical projection neuron (PN) markers and with mature electrophysiological properties. Using *ex vivo* transplantation to human organotypic cultures, we found that the hES-iNs could integrate into adult human cortical networks. We obtained no evidence that the hES-iNs had acquired a distinct cortical layer phenotype. Instead, our single-cell data showed that the hES-iNs, similar to fetal human cortical neurons, expressed both upper and deep layer cortical neuronal markers. Taken together, our findings provide evidence that TF programming can direct human ES cells towards cortical neurons but that the generated cells are transcriptionally profiled to generate both upper and deep layer cortical neurons. Therefore, most likely additional cues will be needed if these cells should adopt a specific cortical layer and area identity.

## Introduction

The human cortex is affected by several debilitating acute and chronic neurodegenerative disorders such as stroke, traumatic brain injury, amyotrophic lateral sclerosis and Alzheimer’s disease, which target specific types of cortical neurons. Emerging evidence indicates that stem cells and reprogrammed cells can be used to generate human cortical neurons both for cell replacement by transplantation, and for disease modeling and drug screening [1, 2]. Several laboratories have established *in vitro* protocols for the derivation of excitatory pyramidal neurons, the principal type of neuron in the adult cortex, from human pluripotent stem cells (hPSCs) [3–5]. Efficient production of corticofugal projection neurons (CfuPNs) from ES cells has also been reported [5]. While the temporal generation of neurons belonging to the different cortical layers is largely maintained *in vitro*, and the presence of neurons belonging to specific layers has been found, the proportion of cells characteristic of each layer varies considerably depending on the method used [6]. In addition, most available protocols are time-consuming, inefficient and the generated neurons are often immature and with incomplete functional properties. It also remains to be assessed how closely the ES cell-derived cortical neurons resemble their *in vivo* counterparts.

Transcription factor (TF) programming is a fast and efficient approach for generating different types of cells. This methodology is based on the logic of direct conversion, using lineage-specific TFs to drive differentiation, but applying them to pluripotent stem cells rather than to somatic cells such as fibroblasts. Transcription factor programming of human ES cells efficiently gives rise to functional excitatory [7] and inhibitory neurons [8]. These human ES cell-derived induced neurons (hES-iNs) exhibit neuronal morphology and gene expression profile, are able to produce action potentials and establish synaptic connections, and survive transplantation into neonatal mouse brain. However, even though the excitatory hES-iNs possess a homogenous gene expression profile resembling that of excitatory forebrain neurons, it is unclear whether they represent a cell population with specific cortical layer and area identity.

Our long-term goal is to develop strategies for efficient production of functional human cortical PNs with specific layer identity using TF programming of ES cells. For this purpose, we have, in this study, chosen to evaluate, in transcription factor programming experiments, the most prominent TFs involved in upper and deep layer PN specification during cortical development. First, SATB2 which represses subcerebral features in callosal neurons, therefore driving upper layer cortical identity [9]. Second, FEZF2 which is a key regulator in deep-layer cortical neuron development [10–12]. We tested SATB2 and FEZF2 in combination with NGN2, a key TF for excitatory cell derivation [13]. The properties of the hES-iNs derived by three different combinations of TFs, i.e., NGN2 only (N), NGN2 plus FEZF2 (NF), and NGN2 plus SATB2 (NS), were analyzed and compared with those of fetal and adult human cortical neurons.

We show here that all three TF combinations were able to drive human ES cells to a neuronal fate, exhibiting properties of functional excitatory cortical neurons, which morphologically resembled adult more closely than fetal human cortical neurons. Using *ex vivo* transplantation to human organotypic cultures, we obtained evidence that these hES-iNs integrated into adult human cortical neural networks. However, immunohistochemistry and patch-clamp electrophysiology showed only subtle differences between the TF combinations in the phenotype of the hES-iNs. This finding was corroborated by single-cell analysis, which also revealed that individual hES-iNs expressed markers of both upper and deep cortical layers, similar to fetal human cortical neurons, but exhibited a more mature neuronal gene expression pattern compared to the fetal cortical cells. Thus, we show that programming using three different TF combinations gives rise to similar progeny, i.e., cells with many properties characteristic of human cortical neurons but lacking the molecular signature signifying specific layer identity.

## Materials and methods

Human fetal tissue was obtained with informed consent from patients from Lund and Malmö University Hospitals according to guidelines approved by the Lund-Malmö Ethical Committee, Sweden (Dnr. 6,1,8-2887/2017). Adult human cortical tissue was obtained with informed consent from patients or LAR/guardians of patients undergoing elective surgery for temporal lobe epilepsy according to guidelines approved by the Regional Ethical Committee, Lund (Dnr. H15 642/2008). All animal related procedures in the present study were conducted in accordance with the European Union Directive (2010/63/EU) on the subject of animal rights, and were approved by the committee for the use of laboratory animals at Lund University and the Swedish Board of Agriculture (Dnr. M68-16).

### Cell culture

Human ES cells H1 (WA01) from WiCell Research Institute (Wicell, WI) were cultured in feeder-free conditions on MATRIGEL^TM^ (BD Biosciences)-coated 6-well plates in mTeSR^TM^1 medium (StemCell Technologies) that was changed daily. Cells were dissociated with Accutase (ThermoFisher Scientific) after reaching 80% confluence and replated in mTeSR^TM^1 medium. Human H1 ES cells were kept in culture and used for all induction experiments between passages 43 to 51.

Mouse glia cells were cultured from the forebrain of newborn wildtype CD1 mice as described [14]. Briefly, newborn mice (P3-P5) were decapitated with sterile scissors, forebrain pieces of newborn mice (P3-P5) were cut by a sterile surgical blade in small pieces subsequently digested with dissociation medium (DM) (0.7 mg/ml hyaluronidase, 0.2 mg/ml kynurenic acid, 1.33 mg/ml trypsin (Sigma) in Hank’s balanced salt solution (HBSS) (Life Technologies)) for up to 30 min. Cell dissociation was facilitated by harsh trituration to avoid contamination of neurons. Cell suspension was then plated onto T75 flasks coated with poly-L-lysine (PLL) (Sigma) in DMEM/F12 supplemented with 10% fetal bovine serum (FBS) (Life Technologies). After reaching confluence, mouse glia cells were dissociated with trypsin and passaged at least 3 times to remove potential trace amounts of neurons before co-culture experiments.

Primary fetal human cortical cells were derived from cerebral cortex of aborted human fetuses (7.2 and 11 weeks of age) according to guidelines approved by the Lund/Malmö Ethical Committee. The tissue was carefully dissected, minced into small pieces and then triturated with pipette tip into a single cell suspension. The cells were either used directly for sorting experiments (fresh fetal human cortical cells, hereafter called hFCtx^F^ cells) or washed with culture medium and plated onto poly-D-lysine (PDL) (Sigma-Aldrich)/Fibronectin (Life Technologies) (both 10μg/mL)-coated glass coverslips at a density of 20 000 cells per cm^2^ and maintained in culture medium (cultured human fetal cortical cells, hereafter called hFCtx^C^ cells). The hFCtx^C^ cells were fixed with 4% paraformaldehyde (PFA) after 3 weeks for immunohistochemistry and morphological analysis.

Adult human cortical tissue was obtained by informed consent from patients undergoing elective surgery for temporal lobe epilepsy (3 females and 1 male, aged 27–49 years) according to guidelines approved by Lund/Malmö Ethical Committee. Primary adult human cortical cells (hACtx^C^) and slice cultures (hACtx^S^) were derived subsequently. For the hACtx^C^ cultures, the tissue samples were submerged in Hibernation medium (Thermo Fisher Scientific, MA, USA) immediately after surgery and transferred to a cell culture laboratory. The meninges were removed under a dissection stereomicroscope (Leica, Germany) and the tissue was cut into small pieces with a sterile surgical blade under a laminar flow bench. The tissue was then processed using a kit for dissociation of adult brain tissue (Neural Tissue Dissociation Kit, Miltenyi, Germany) according to the manufacturer’s instructions. A single cell suspension was obtained and plated onto PDL- (Sigma Aldrich, USA) and human fibronectin- (Thermo Fisher Scientific, USA) coated culture flasks (Nunc, Thermo Fisher Scientific, USA), glass/plastic chamberslides and glass/plastic Petri dishes (all from Ibidi, Germany). The cells were cultured in Neurobasal medium supplemented with B27 (1:50) and Glutamax (1:100) (Thermo Fisher Scientific) and recombinant human factors such as: Brain Derived Neurotrophic Factor (hBDNF), Glial cell Derived Neurotrophic Factor (GDNF) and Ciliary Neurotrophic Factor (CNTF) (10 ng/ml) (all from Peprotech, UK).

#### Virus production and induction of cortical neurons

All lentiviral vectors were handled in a class II biosafety laboratory. Lentiviral particles with the VSVG capsid were prepared according to Dull and co-workers [15]. The human consensus coding sequences (CCDS) of NEUROGENIN2 (henceforth referred to as NGN2), FEZF2 and SATB2 were synthesized (Genscript, CA) and cloned into EcoRI/BamHI site of tetO-FUW lentiviral vectors carrying resistance genes for blasticidin (Addgene plasmid #97330) and puromycin (Addgene plasmid #97329). The rtTA expressing lentiviral vector (Addgene plasmid #20342) was used along with vectors mentioned above to induce cortical fate in H1 ES cells. Cells were additionally transduced with FUW-tetO-GFP lentiviral vector before co-culture with adult human cortex organotypic slices.

On day -2, H1 cells were passaged using Accutase and 5x10^5^ cells re-plated on MATRIGEL^TM^-coated 6-well plates in mTeSR^TM^1 medium with Rock Inhibitor (10 μM, Y-27632, StemCell Technologies). The day after (day -1), medium was replaced with fresh mTeSR^TM^1 containing polybrene (4 μg/ml, Sigma-Aldrich) and 1μL of each virus was added per well. One day after infection (day 0), medium was replaced with induction medium (DMEM/F12 (Life Technologies), supplemented with N2 (100x, Invitrogen). Doxycycline (2.5 μg /ml, Sigma) was added on day 0 to induce TetO gene expression, and kept in the medium until the end of experiment. On day 1, a 6-day puromycin (1.25 μg/ml) and blasticidin (1.25 μg/ml, Invitrogen) selection period was started. On day 7, cells were dissociated with Accutase and replated onto poly-L-Ornithine (10 μg/ml, Sigma)–Laminin (10μg/ml, Invitrogen) coated glass coverslips in 24 well plates, along with mouse glia in maturation medium (Neurobasal, Life Technologies) supplemented with B27 (50x, Invitrogen) and Glutamax (250x, Invitrogen), containing BDNF (10 μg/ml, PeproTech). 5-Fluoro-2’-deoxyuridine (5-FUdR) (2 mg/ml) was added to the medium to inhibit glia proliferation on day 2 of co-culture. FBS (2.5%) was added on day 7 of the co-culture to support glia viability. 50% of medium was replaced every 2–3 days, and cells were kept in co-culture for up to 8 weeks.

#### Co-culture of hES-iNs with adult human cortical slices

Adult human cortical slices were derived and handled as previously described [16]. Briefly, the surgically resected tissue was immediately kept in ice-cold modified human artificial cerebrospinal fluid (mhACSF) and then glued to the slicing stage within the chamber of a Vibratome (Leica VT1200S) filled with ice-cold mhACSF. 300 um thickness slices were cut and kept in 24-well plates containing ice-cold mhACSF with one slice per well until the sectioning was completed. The slices were then transferred to cell culture inserts containing Alvetex scaffold membranes (Reinnervate) in 6-well plates filled with slice culture medium (BrainPhys medium, Stemcell) supplemented with 2% B27, Glutamax (1:200), Gentamycin (50 ug/ml) (Life Technologies)) and incubated in 5% CO2 at 37°C. Medium was changed once a week. FUW-TetO-GFP transduced hES-iNs were plated on top of the slices at 5 days after the start of transgene induction, and co-cultured for another four weeks before they were either fixed in 4% PFA and assessed by immunohistochemistry, or used for electrophysiological recordings and subsequently fixed and stained.

### Single cell qPCR

Candidate genes related to cortical layer or region identity and neuronal function were selected from multiple sources [16–20]. UBC and YWHAZ were included as housekeeping genes. Control genes normally expressed in dopaminergic, noradrenergic, serotonergic and hindbrain neurons were also included. A complete list of TaqMan assays is shown in Table 1.

**Table 1:** List of primers/probes used in single cell qPCR analysis.

Target	Alias	Expression / Function in	Assay ID
MAP2		pan-neuronal	Hs00258900_m1
TUBB3	TUBULIN, beta	pan-neuronal	Hs00964963_g1
PAX6		neural progenitors	Hs00240871_m1
NEUROD1		telencephalon	Hs00159598_m1
NEUROG2^*^		neural progenitors	Hs00702774_s1
FOXG1^*^		telencephalon	Hs01850784_s1
EMX2		neural progenitors	Hs00244574_m1
NR2F1	COUPTF1	temporal lobe (8–12 pcw)	Hs01354342_mH
CUX1	CUTL1	progenitor cells and upper-layer neurons	Hs00738851_m1
LIMCH1		layer 1–2 CPN	Hs00405524_m1
NECTIN3		layer 1–2 CPN	Hs00210045_m1
INHBA		layer 1–3 CPN	Hs00170103_m1
LMO4		layers 2/3 and 5, CPN	Hs01086790_m1
CHN2		layer 3 CPN	Hs00906969_m1
LPL		layer 4 CPN	Hs00173425_m1
EAG2	KCNH5	layer 4 (upper) CPN	Hs00544949_m1
RORB		layer 4 (lower) CPN	Hs00199445_m1
SATB2		upper layers and some layer 5 neurons & neocortex	Hs01546828_m1
POU3F2	BRAIN2	progenitor cells and upper-layer neurons	Hs00271595_s1
ETV1		layer 5 cortical-striatal PN	Hs00951951_m1
BHLHE22^*^	BHLHB5	cortical-spinal/motor neurons	Hs01084964_s1
DIAPH3		cortical-spinal/motor neurons	Hs01107330_m1
CNTN6		layer 5b cortical-spinal/motor neurons	Hs00274291_m1
PCP4		layer 5b cortical-spinal/motor neurons	Hs01113638_m1
S100A10		layer 5	Hs00741221_m1
SOX5^*^		cortical-spinal/motor neurons	Hs00753050_s1
CRYM		deep-layer subcerebral PN	Hs00157121_m1
FEZF2		telencephalon	Hs01115572_g1
BCL11B	CTIP2	deep-layer neurons	HS00256257_m1
TBR1		layer 1 and deep-layer neurons & telencephalon (8–12 pcw)	Hs00232429_m1
TLE4		deep-layer & CThPN	Hs00419101_m1
FOXP2		layer 6 CThPN	Hs01074133_m1
FOG2	ZFPM2	layer 6 CThPN	Hs01101779_m1
PLEXIND1	PLXND1	layer 5 CPN	Hs00892417_m1
DKK3		layer 6 CPN	Hs00247429_m1
NR4A2	NURR1	dopaminergic/midbrain	Hs00428691_m1
TH		dopaminergic/midbrain	Hs00165941_m1
TPH1		serotonergic	Hs00188220_m1
HTR2C		serotonergic & layer 5	Hs00168365_m1
LBX1		hindbrain	Hs00198080_m1
FOXO1		layer 5b & hindbrain	Hs01054576_m1
DBH		adrenergic	Hs01089840_m1
SLC17A7	VGlut1	glutamate transport	Hs00220404_m1
SLC17aA6	VGlut2	glutamate transport	Hs00220439_m1
GAD1	GAD67	GABA synthesis	Hs01065893_m1
GAD2	GAD65	GABA synthesis	Hs00609534_m1
UBC	UBIQUITINC	control	Hs00824723_m1
YWHAZ		control	Hs03044281_g1

pcw–post conception week

* These primers/probes detect endogenous gene expression as well as transgene expression.

Single-cell suspension was generated using Accutase dissociation followed by labeling for Neural Cell Adhesion Molecule (NCAM) and CD44 (BD Biosciences) as previously described [16]. Cells were sorted on a FACSAriaII cell sorter (BD Biosciences). Zero-, 10- and 50-cell controls were included to control for gene amplification specificity. Gates were set to include only live (Draq7 (Abcam) negative) cell populations. Single NCAM^+^ cells were sorted into 96-well PCR plates containing lysis buffer (0.4% NP40, deoxynucleoside triphosphates, dithiothreitol, and RNase OUT (Invitrogen)) and snap frozen on dry ice. Upon thawing, CellsDirect reaction mix containing SSIII/PlatinumTaq (CellsDirect One-Step RT_qPCR kit, no ROX, Invitrogen) and the selected 48 TaqMan assays at a final dilution of 0.05× were added to the cell lysate for RT-PCR pre-amplification. RT–PCR pre-amplification cycling conditions were: 50°C, 60 min; 95°C, 2 min; 25×(95°C, 15 s; 60°C, 4 min). Next, individual TaqMan assays were combined with assay loading reagent (Fluidigm) before being pipetted onto a Biomark chip (Fluidigm). The cDNA from the 96-well PCR plates was diluted with water 1 in 5, combined with sample loading reagent (Fluidigm) and loaded onto the same Biomark chip. This was run on a Biomark analyser system (Fluidigm). The arrays were read in a Biomark genetic analysis system (Fluidigm) and the data exported to Microsoft Excel for downstream analysis. The amplification curves were quality-controlled and data filtered according to no-reverse-transcription control reactions and to exclude data with Ct>25. Detection thresholds [21] were automatically generated using a baseline linear correction model and a quality threshold of 0.65. A well was defined as containing a cell that had been successfully reverse transcribed if there were detectable levels (Ct < 25) of at least one out of two housekeeping genes. Genes detected in no template controls were excluded from further analysis.

We used hES-iNs sorted from 2 independent viral transduction experiments and ran single cell qPCR analysis on 6 chips of 48x48 (Fluidigm).

### Immunocytochemistry

Cultures were washed with phosphate-buffered saline (PBS) and fixed in 4% PFA for 20 min. The coverslips were washed three times with PBS, permeabilized with 0.025% TritonX-100 in PBS for 10 min, and then blocked with 5% normal donkey serum (NDS) for 45 min at room temperature. Primary antibodies, mouse anti-MAP2a/b (MAP2; 1:1000, Sigma) or chicken anti-MAP2 (1:5000, Abcam), mouse anti-SATB2 (1:100, Abcam), goat anti-BRAIN2 (1:400, SantaCruz), rabbit anti-RFP (1:1000, Abcam), and rabbit anti-TBR1 (1:1000, ProteinTech), diluted in blocking solution were applied overnight at 4°C. For SATB2 and BRAIN2 immunostaining, antigen retrieval with 10mM Citrate buffer (pH 6.0) was performed for 30 min, 65°C. After three rinses with PBS, Alexa Fluor 488, Cy3 and Alexa Fluor 647 conjugated donkey or goat secondary antibodies (1:800, Jackson Immunoresearch, PA) against the respective primary antibodies diluted in blocking solution were applied for 1.5 h. Streptavidin-conjugated Alexa Fluor 647 was used for visualizing cells filled with biocytin from electrophysiology experiments. Cell nuclei were counterstained with Hoechst33342 and mounted with polyvinyl-alcohol-1,4-diazabicyclo(2.2.2)octane (PVA-Dabco).

For the adult human cortical slice cultures and their co-cultures with hES-iNs, slices were fixed in 4% PFA overnight and then rinsed three times in KPBS for 15 min. The slices were then permeabilized in potassium-PBS (KPBS) with 0.02% bovine serum albumin (BSA) and 1% Triton X-100 at 4°C over night, blocked in KPBS with 0.2% Triton X-100 and 1% BSA and 5% of NDS or normal goat (NGS) sera at 4°C over night. After the blocking step, slices were incubated for 48 h with primary antibodies and then for an additional 48 h with respective secondary antibodies in blocking solution at 4°C. The slices were then washed three times in KPBS and incubated in Hoechst 33342 for 2 h at room temperature. Slices were mounted on glass slides with PVA-Dabco after rinsing with deionized water.

### Microscopy

Images were obtained on BX61 epifluorescence (Olympus, Japan) and LSM780 confocal (Zeiss, Germany) microscopes.

#### Pyramidal morphology index

The pyramidal morphology index (PMI) was defined as the ratio between the width of the largest process and the total number of processes crossing a sampling circle [22] in CellSens imaging software (Olympus, Japan). To determine the PMI, at least 25 N, NF and NS-induced hES-iNs from at least 3 independent induction experiments, and cultured fetal and adult human cortical neurons were randomly chosen. The number of processes crossing the sampling circle was counted and the width of the widest neurite was measured. The index was calculated for multipolar cells that had more than two processes.

#### Cell soma size

The cell soma size was measured from 25 cells randomly chosen from at least 3 independent induction experiments of N, NF and NS-induced hES-iNs and cultured fetal and adult human cortical neurons in CellSens imaging software. Images were obtained using 20X objective and the outline of the cell body was delineated manually. The area corresponding to the cell soma was calculated by the software and used for analyzing the average cell soma size.

#### Neuronal morphology tracing

Neuronal morphology was assessed for 3 representative N, NF and NS-induced hES-iNs and cultured fetal and adult human cortical neurons using simple neurite tracer plugin in Fiji [23]. The outlined areas were filled out and then converted to 8-bit monochrome images in Fiji.

### Electrophysiology

Whole-cell patch-clamp recordings were performed with a HEKA double patch clamp EPC10 amplifier using PatchMaster for data acquisition. Cells were grown on coverslips and transferred to the recording chamber. The coverslip was constantly perfused (1ml/min) with carbogenated artificial cerebrospinal fluid (aCSF, in mM: 119 NaCl, 2.5 KCl, 1.3 MgSO_4_, 2.5 CaCl_2_, 26 NaHCO_3_, 1.25 NaH_2_PO_4_, and 11 glucose, pH 7.2–7.4, 295–300 mOsm) at 34°C. Recording pipettes were filled with intracellular solution containing (in mM): 122.5 KGlu, 12.5 KCl, 10.0 HEPES, 8.0 NaCl, 2.0 Na_2_ATP, and 0.3 Na_2_GTP for recordings of intrinsic properties and for detection of spontaneous activity in hES-iNs transplanted onto organotypic slices and 135 CsCl, 10 HEPES, 10 NaCl, 2 Na_2_ATP, and 0.3 NaGTP for recordings of sPSC. Intracellular solutions had pH of 7.2–7.4, osmolarity of 285–295 mOsm, and resistance of 2–12 MΩ. 2–4 mg/ml biocytin were added to the internal solution prior recording for *post hoc* identification of the recorded cell. 5 mM QX314 was added to the internal solution before recording of sPSCs. Voltage values were not corrected for the liquid junction potential, which was 13.82 mV and 5.10 mV for KGlu-, CsCl-based internal solutions, respectively. Only cells with a series resistance below 30 MΩ were included in the analysis.

Voltage- and current-clamp recordings were used for electrophysiological characterization. Sodium and potassium currents were evoked by a series of 200 ms long voltage steps (from -70 mV to +40 mV in 10 mV steps) and their sensitivity to 1 μM tetrodotoxin (TTX) and 10 mM tetraethylammonium (TEA), respectively was determined. A series of current steps (0–200 or 400 pA in 10 pA steps) lasting 500 ms were performed from a membrane potential of ~-70mV (current was injected when needed to keep the membrane potential ~-70mV) to determine the cell’s ability to generate action potentials (APs). Spontaneous postsynaptic currents (sPSCs) were recorded from a holding potential of—70 mV. AMPA and NMDA receptors were blocked by 5 μM 2,3-Dioxo-6-nitro-1,2,3,4-tetrahydrobenzo[*f*]quinoxaline-7-sulfonamide (NBQX) and 50 μM D-(-)-2-Amino-5-phosphonopentanoic acid (D-APV), respectively. GABA receptors were blocked by 0.1 mM picrotoxin (Ptx). Data were analyzed offline with Fit-Master, IgorPro 6.3 and NeuroMatic v2.8b.

### Data representation and statistics

Statistical comparisons were performed using GraphPad Prism version 6.05 (GraphPad software, La Jolla, CA), by Student’s t-test, Mann-Whitney test or one-way ANOVA. Data were presented as means ±SEM, and differences were considered significant at p < 0.05.

## Results

### Three transcription factor combinations generate cortical-like neurons from human ES cells

**Fig 1:**
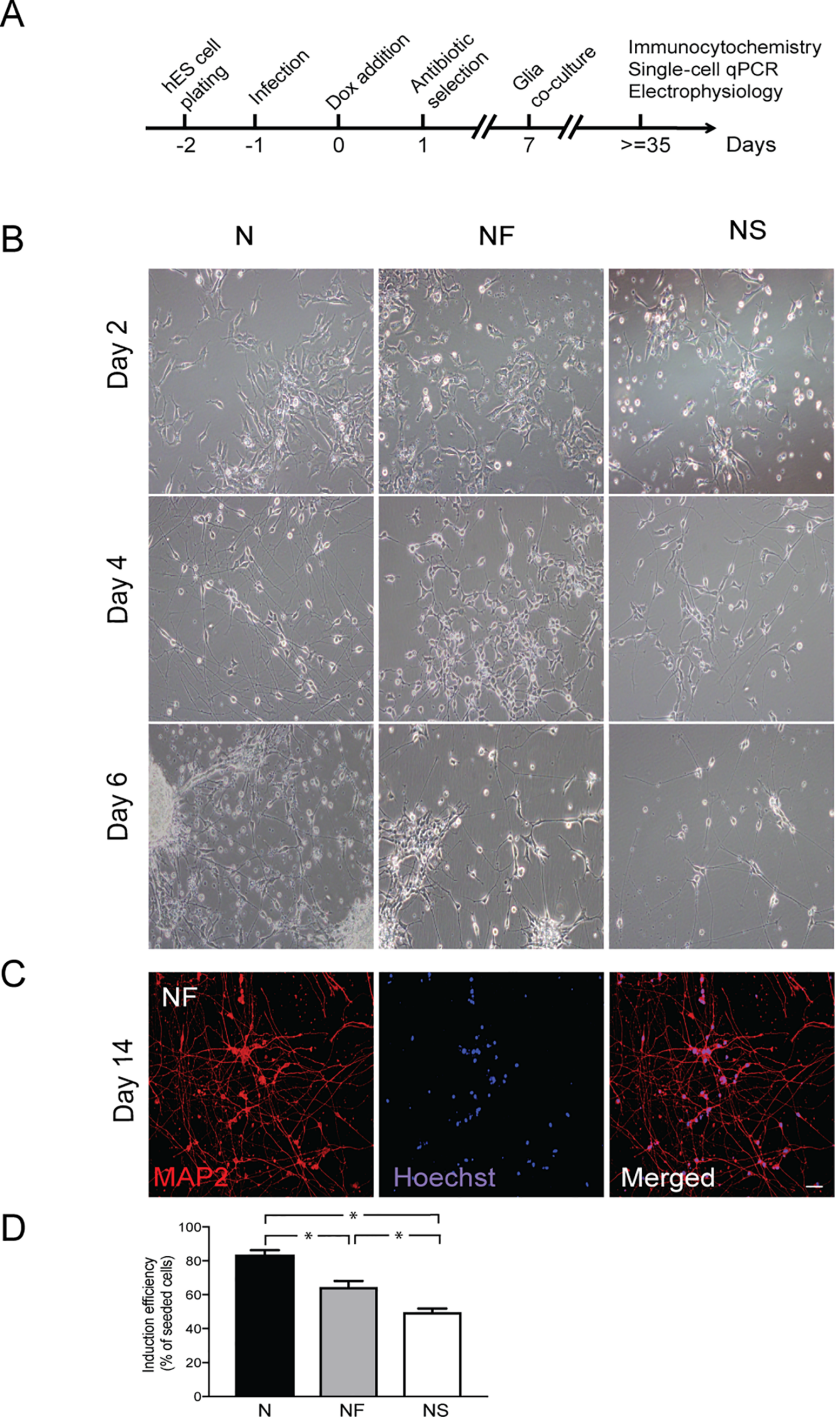
Three TF combinations rapidly induce neuronal cells from ES cells. **(A)** Experimental design of hES-iN generation. **(B)** Representative images illustrating time course of generation of hES-iNs from ES cells by NGN2 only (N), NGN2 plus FEZF2 (NF) or NGN2 plus SATB2 (NS). Note that hES-iNs in all three TF combinations change their morphology from flat to more round with thin neuron-like projections developing already at day 4 after induction. **(C)** Representative images of hES-iNs (NF combination) at day 14 of induction protocol. Cells have already formed long neuronal projections as well as complex neuronal networks. For hES-iNs generated with N and NS, see S1A Fig. **(D)** Neuronal yield at 14 dpi (three independent differentiation experiments), induced by the different transcription factor combinations. Mean±SEM; *, p<0.05, one-way ANOVA. Scale bar: 50 μm.

Forced expression of NGN2 rapidly and efficiently drives human ES cells to form excitatory neurons with telencephalic properties [7]. In an attempt to specify the fate of these NGN2-induced cells towards specific cortical neuronal subtypes, we combined NGN2 with either SATB2 or FEZF2. Similar to previous studies [24, 25], we delivered the TFs to the human ES cells by doxycycline (Dox)-inducible lentiviral vectors carrying TFs coupled to resistance genes, along with a vector for constitutive expression of rtTA. Following the standard protocol described by Zhang and colleagues [7] (Fig 1A), the human ES cells were plated and infected with lentivirus the following day. Expression of TFs was induced by Dox 24 h later. After a 7-day antibiotic selection period, newly formed hES-iNs were plated onto mouse glia to support neuronal maturation and synapse formation in long-term culture (Fig 1A).

Already on the second day of induction, the human ES cells no longer formed colonies (Fig 1B) or proliferated but started to rapidly extend processes and form round soma, exhibiting neuron-like morphology. All combinations of TFs had produced neuron-like cells by the end of the first week. Using immunocytochemistry, we found that NGN2 alone or in combination with SATB2 or FEZF2 gave rise to cells with mature neuronal morphology expressing MAP2 at two weeks (Fig 1C and S1A Fig). We observed a slightly lower yield, calculated as a function of the starting number of human ES cells, in NF- and NS- (64% and 49%, respectively) (Fig 1D) as compared to N-derived hES-iNs (83%). This difference was most likely caused by the antibiotic selection step. Thus, in the NF and NS conditions the human ES cells had to be transduced with two types of viral particles in order to survive the antibiotic selection step. This allowed enrichment of double-transduced cells in hES-iN cultures but reduced the overall neuronal yield.

**Fig 2:**
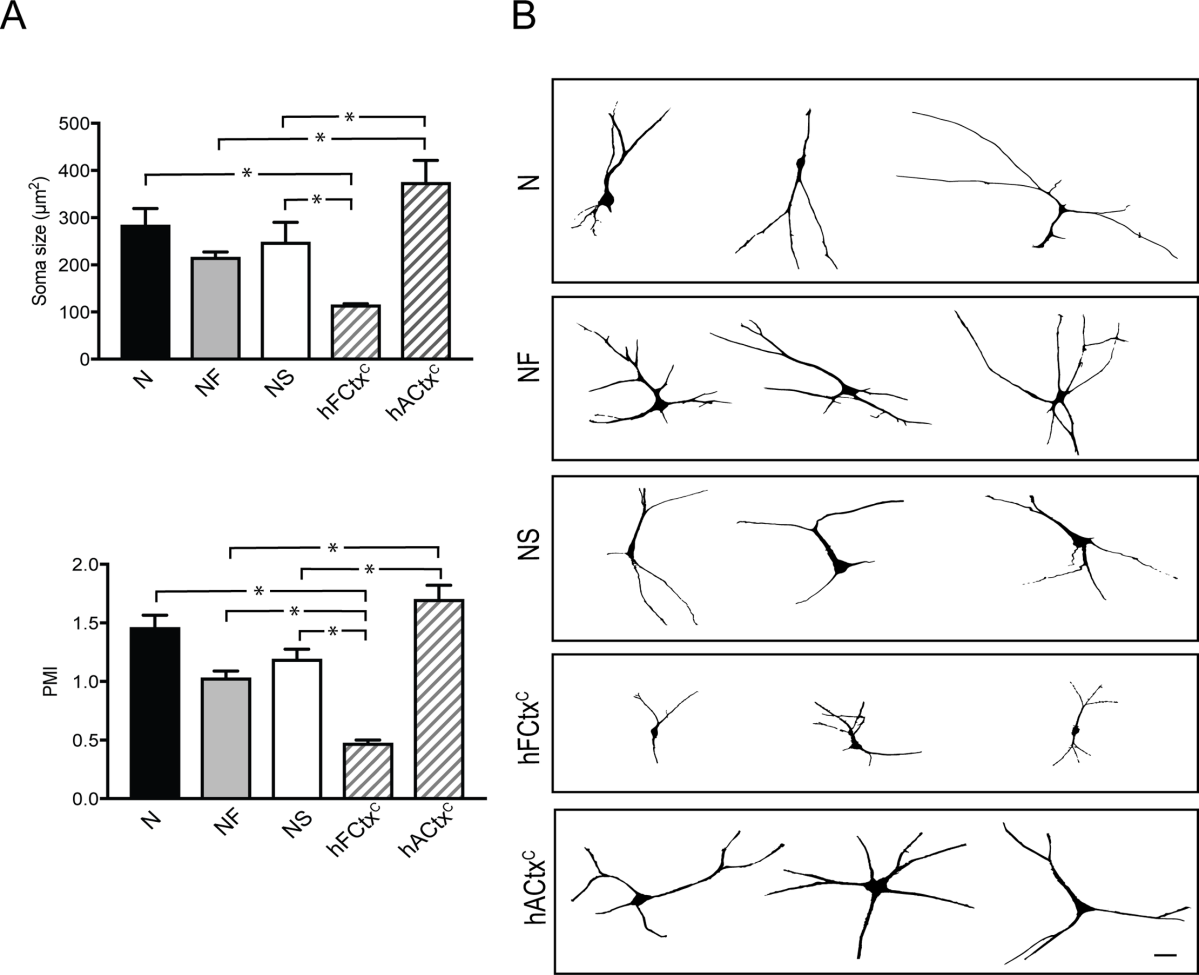
Three TF combinations induce pyramidal-shaped neurons morphologically resembling adult human cortical neurons. (A) Bar diagrams illustrating soma size (*top*) and pyramidal morphology index (PMI, *left*) of hES-iNs compared to hFCtx^C^ and hACtx^C^ cells. Mean±SEM (n = 3); *, p<0.05, one-way ANOVA. (B) Tracing showing the pyramidal shape and complex morphology of the hES-iNs at 8 weeks in culture, different from the hFCtx^C^ cells but resembling that of hACtx^C^ cells. Scale bar = 20 μm; n corresponds to a number of independent differentiation experiments.

Next, we analyzed morphology and soma size as well as the expression of cortical neuronal markers in the hES-iNs co-cultured with mouse astrocytes for 8 weeks. We compared hES-iNs with cultured fetal (hFCtx^C^) and adult human cortical (hACtx^C^) neurons. Morphometric measurements revealed that all hES-iNs generated with the NF combination had soma size similar to that of hACtx^C^ cells, which surpassed that of hFCtx^C^ neurons (Fig 2A). The morphological complexity was then determined using the pyramidal morphology index (PMI), an unbiased numerical characterization of dendritic morphology which takes into account the number of neurites emerging from the cell body and the thickness of the apical dendrite [22]. We did not observe any differences between N-, NF- and NS-derived hES-iNs in PMI (Fig 2A). The PMI of hES-iNs derived using any of the 3 combinations was higher than for hFCtx^C^ neurons, but only the N-condition gave rise to neurons which did not differ in their PMI as compared to hACtx^C^ (Fig 2A). Most hES-iNs were pyramidal-shaped with rich arborizations comparable to what was observed with hACtx^C^. Importantly, hFCtx^C^ cells also exhibited pyramidal-shape, but their smaller soma and less complexity of processes as compared to both hES-iNs and hACtx^C^ (Fig 2B) suggested that the human ES-iNs were more mature than the hFCtx^C^ cells.

**Fig 3:**
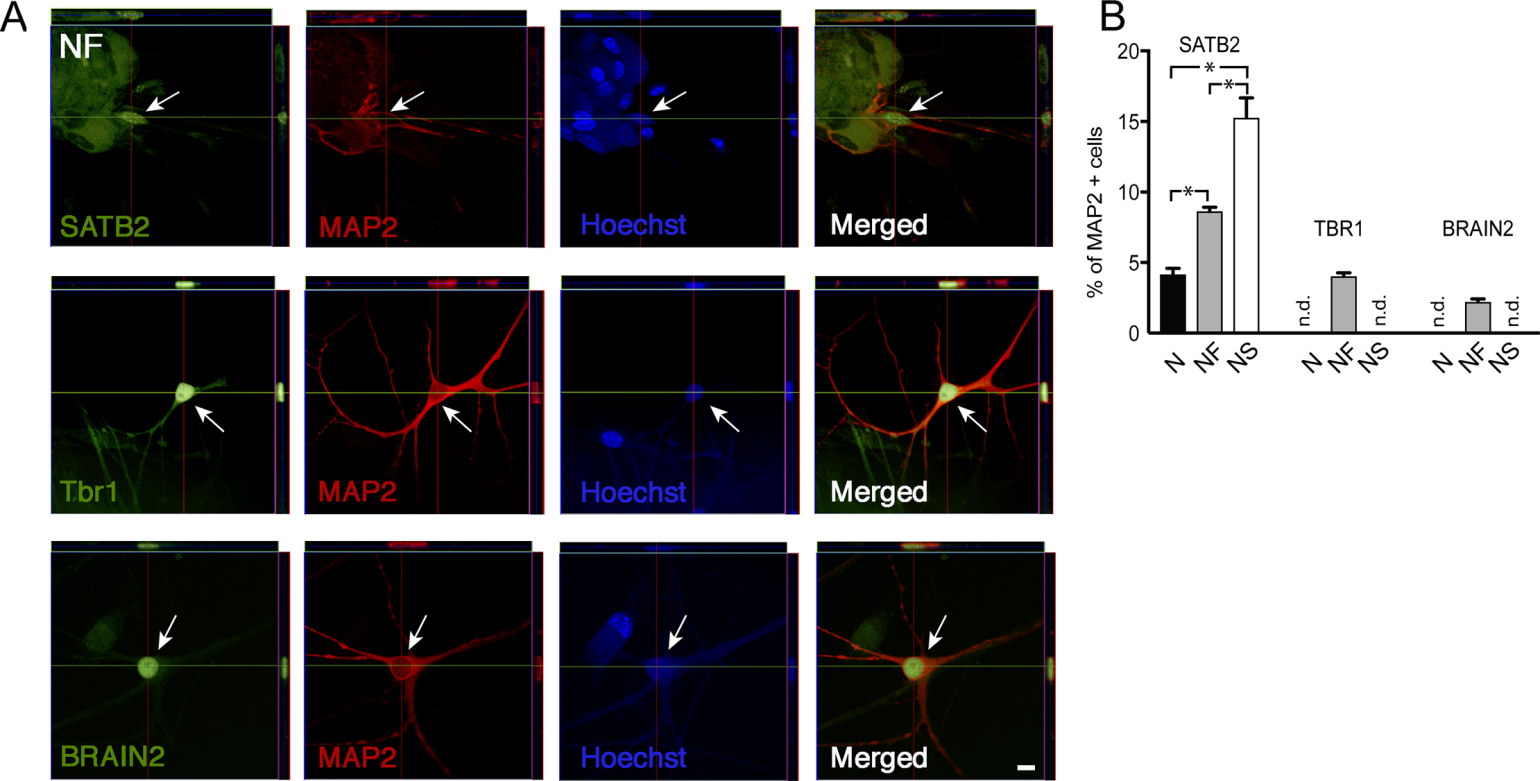
Human ES-derived cortical-like cells express PN markers *in vitro*. (A) Orthogonal reconstructions from confocal z-series showing examples of deep layer marker TBR1 and upper layer markers SATB2 and BRAIN2 in NF-derived hES-iNs. Scale bar = 10 μm. Arrows depict cells expressing the respective markers. **(B)** Quantification of cortical marker expression in hES-iNs derived by three TF combinations as revealed by immunocytochemistry (n = 3). n corresponds to a number of independent differentiation experiments; *, p<0.05, one-way ANOVA. n.d.- not detectable.

Using immunocytochemistry, we found that the percentage of MAP2+ hES-iNs expressing the predominantly upper deep layer callosal projection neuron (CPN) marker SATB2 (Fig 3A) differed between the NS (around 15% SATB2+ cells), NF (9% SATB2+ cells), and N (4% SATB2+ cells) combinations (Fig 3B). Some NF-derived hES-iNs expressed the deep layer marker TBR1 (2%) and some the upper layer marker BRAIN2 (4%) (Fig 3B), but expression of these markers was not detected in either N- or NS-derived hES-iNs. For comparison, we stained hFCtx^C^ cells of an 11-week fetus as well as fresh adult human cortical tissue slices. We found expression of TBR1 and SATB2 in a small population of hFCtx^C^ cells, whereas BRAIN2 was expressed by nearly half of these cells (S1B Fig). In addition, we detected TBR1 but not SATB2 or BRAIN2 expression in the adult human cortical tissue (S1C Fig).

Taken together, our findings provide evidence that the tested combinations of TFs are equally competent to drive human ES cells to form pyramidal-shaped neuron-like cells but that the generated cells show minor differences in the expression of key markers for cortical PNs. Our findings also raise the possibility that the NF combination may have initiated a differentiation program resulting in hES-iNs expressing a wider range of cortical markers as compared to the N and NS combinations. The comparison of hES-iNs with fetal and adult human cortical neurons suggests that the differentiation of hES-iNs is more advanced as compared to that of hFCtx^C^ cells, at least with regard to soma size and complexity of processes.

#### Three transcription factor combinations direct human ES cells to neurons with molecular phenotype of both upper and deep cortical layers

In order to define the molecular phenotype of the hES-iNs generated by the different TF combinations and to assess their degree of heterogeneity, we next performed single-cell gene expression analysis. We quantitatively analyzed the expression of 48 genes (Table 1) involved in cortical development as well as known markers for specific cortical neuron subtypes at the single-cell level using Fluidigm. We used NCAM as a positive marker to isolate hES-iNs from co-cultures with glia and, for comparison, cortical neurons from a 7-week-old fetal human brain (hFCtx^F^ cells) by FACS.

**Fig 4:**
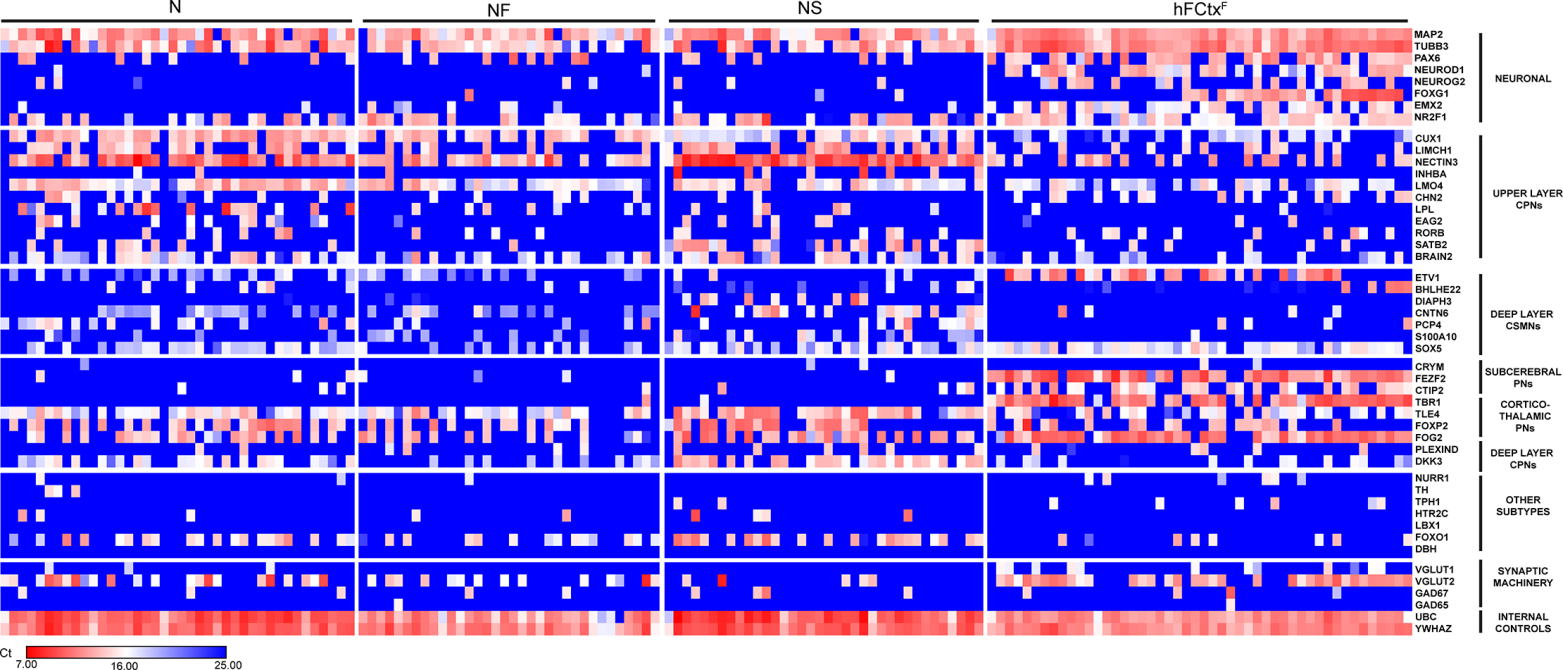
Three TF combinations induce hES-iNs with similar molecular signature resembling that of fetal human cortical neurons. Single-cell quantitative RT-PCR analysis of expression levels of different cortical genes listed to the right. Expression levels are shown as Ct values and color-coded as shown on the bottom. The mRNA levels were quantified in hES-iN cells, co-cultured with primary mouse astrocytes for 8 weeks and FACS-sorted as NCAM+, and in hFCtx^F^ cells from a 7 weeks old human fetus.

Analysis of 189 cells (N: 51; NF: 43; NS: 40; and hFCtx^F^: 55 cells) revealed a complex gene expression pattern in both hES-iNs and hFCtx^F^ neurons. All cells expressed the pan-neuronal markers MAP2 or beta-III-Tubulin as well as the telencephalic marker NR2F1. A majority of hFCtx^F^ cells (79%) and N- and NF-derived hES-iNs (67% and 55%, respectively) expressed the glutamatergic neuronal marker vGLUT2, whereas this marker was detected in fewer NS-derived hES-iNs (15%). Neither hFCtx^F^ cells nor hES-iNs expressed markers of inhibitory (GAD65, GAD67), dopaminergic (NR4A2, TH), serotonergic (TPH1, HTR2C), hindbrain (LBX1), or noradrenergic (DBH) neurons. Importantly, we detected expression of neural progenitor markers such as PAX6, NEUROD1 and EMX2 in hFCtx^F^ cells but not in hES-iNs (Fig 4). This finding provides evidence that the hES-iNs had advanced further into the differentiation process than the hFCtx^F^ cells.

Surprisingly, we found expression of both upper and deep layer cortical markers in single hES-iNs and hFCtx^F^ cells. Upper layer CPN markers such as CUX1, LMO4, NECTIN3, and BRAIN2 were present in most hFCtx^F^ cells (53%, 71%, 51%, 42%, respectively) and hES-iNs (N: 90%, 96%, 90%, 76%; NF: 79%, 91%, 84% 58%; and NS: 85%, 83%, 100%, 65%). The hES-iNs expressed CUX1 and NECTIN3 at relatively higher levels than the hFCtx^F^ cells. All analyzed cell populations also shared a rather similar level of expression of the deep-layer cortico-spinal PN marker SOX5 (hFCtx^F^: 91%; N: 75%; NF: 44%; and NS: 70%). We found expression of the cortico-striatal PN marker ETV1 and the subcerebral PN markers FEZF2, CTIP2 (BCL11B), and TBR1 in hFCtx^F^ cells (91%, 93%, 62%, 85%, respectively), but these markers were not detected in hES-iNs. Cortico-thalamic PN markers such as TLE4, FOXP2 and FOG2 were expressed at similar levels in hES-iNs and hFCtx^F^ cells. Interestingly, we detected high relative expression of the deep-layer CPN marker DKK3 in many hES-iNs generated by the N and NS combinations (78% and 65%, respectively) but in only 23% of NF-derived hES-iNs. A low relative expression level of DKK3 was observed in a small portion (26%) of hFCtx^F^ cells.

Taken together, our results indicate that the tested TF combinations gave rise to hES-iNs with molecular signature of excitatory cortical neurons. The hES-iNs shared many features of molecular phenotype with hFCtx^F^ cells, including the expression of markers of both upper and deep cortical layers in individual cells. However, the hES-iNs showed a more mature neuronal gene expression pattern, lacking expression of immature markers, as compared to that of hFCtx^F^. This is consistent with our finding that the morphological characteristics of hES-iNs more closely resembled those of hACtx^C^.

#### Three transcription factor combinations generate functional cortical neurons with similar electrophysiological properties

In order to assess the electrophysiological properties of the hES-iNs generated by the different TF combinations, we next performed whole-cell patch-clamp recordings of N-, NF- and NS-derived hES-iNs after 4–5 and 8 weeks of culturing. The averaged resting membrane potential (V_rest_) for the hES-iNs ranged from -35 mV to -59 mV (Table 2). The V_rest_ for N-derived hES-iNs became more hyperpolarized at 8 weeks, as compared to 4 weeks, of culturing, whereas V_rest_ did not change between 4–5 and 8 weeks for NF- and NS-derived hES-iNs (Table 2). This finding suggests that the NF- and NS-derived hES-iNs matured faster than the N-derived hES-iNs, as evidenced by V_rest_ reaching a plateau already after 4–5 weeks of culturing.

**Table 2:** Basic electrophysiological characteristics of hES-iNs *in vitro*.

	N		NF		NS	
	4 weeks	8 weeks	4 weeks	8 weeks	5 weeks	8 weeks
n	6	6	7	6	7	7
V_rest_	-36 ± 3mV	-50 ± 3mV*	-35 ± 3mV	-40 ± 4mV	-59 ± 3mV	-54 ± 5mV
R_input_	866 ± 62MΩ	511 ± 86MΩ*	1320 ± 189MΩ	948 ± 121 MΩ	323 ± 65 MΩ	611 ± 80 MΩ*
C	3.1 ± 0.8pF	31 ± 15pF*	2.8 ± 0.6pF	7.7 ± 2.5pF	18 ± 4.1pF	6.9 ± 0.9pF*

* indicates significant difference between 4–5 and 8 weeks.

**Fig 5:**
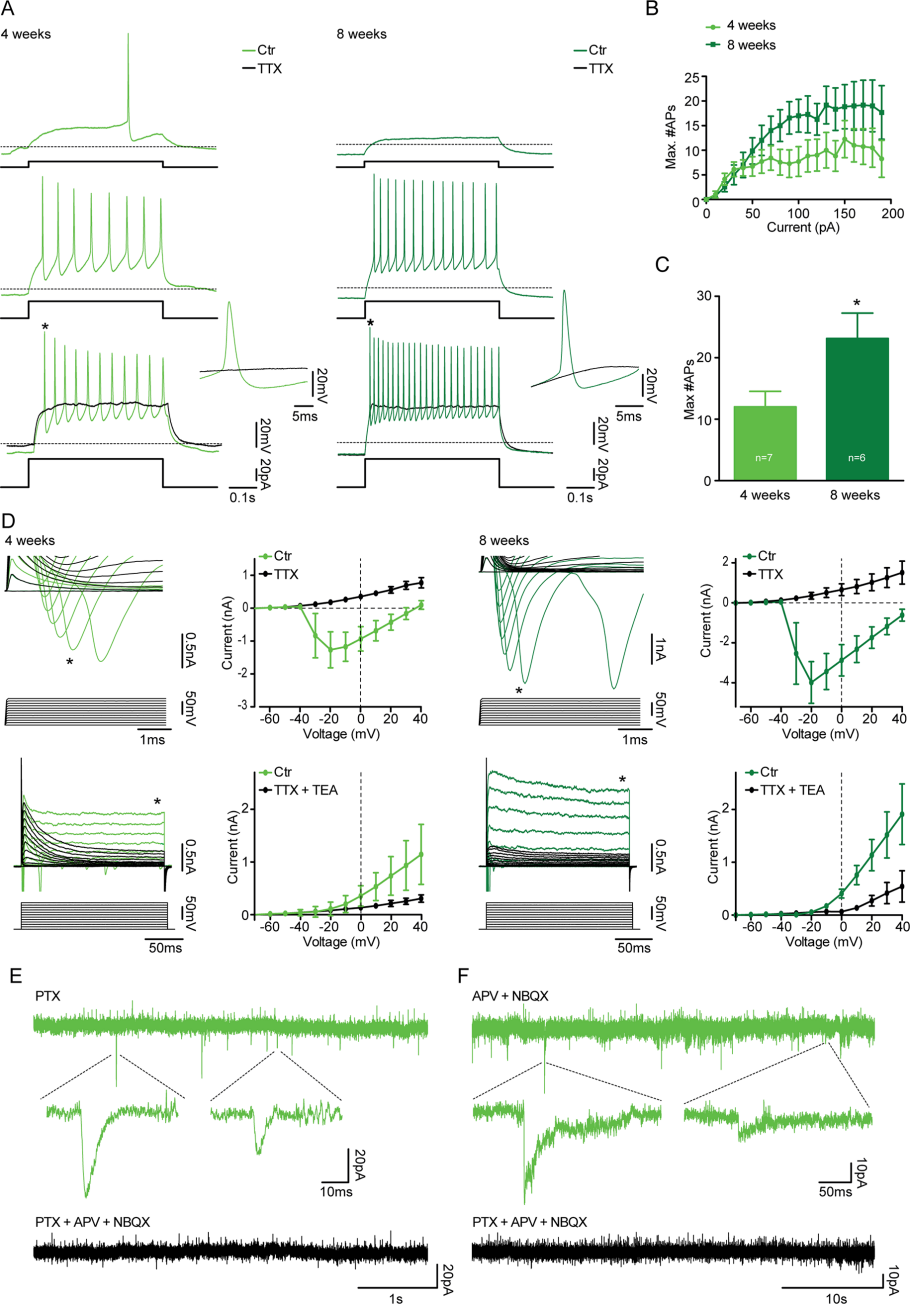
Three TF combinations induce hES-iNs which form functional synapses. Whole-cell patch-clamp recordings were performed from NF-derived hES-iNs at 4 (n = 7) and 8 (n = 6) weeks after induction. For hES-iNs generated with N and NS, see S2 and S3 Figs. **(A)** Representative voltage traces illustrating the NF-derived hES-iNs’ ability to generate APs during a current step from a holding potential of -70mV at 4 (light green) and 8 (dark green) weeks after induction. APs were completely abolished in the presence of 1μM TTX (black traces). * indicates expanded APs. **(B)** Diagram illustrate the number of APs generated plotted against the current steps. **(C)** Bar diagram illustrate the maximal number of APs generated during current steps (10–190 pA in 10 pA steps). * indicates significant difference (p < 0.05). **(D)** Expanded current traces illustrate the inward sodium current (*top*, denoted by *) and the outward sustained potassium current (*bottom*, denoted by *) activated during voltage steps ranging from -70 mV to +40 mV in 10 mV steps at 4 (light green) and 8 (dark green) weeks after induction. The sodium and the potassium current were blocked by the presence of 1 μM TTX (*top*, black) and 1μM TTX + 10 mM TEA (*bottom*, black), respectively. The plots illustrate the sodium current peak (*top*) and the outward potassium current (*bottom*) plotted against the voltage steps in the absence and presence of TTX and TTX+TEA, respectively. **(E)** Current trace illustrates the presence of glutamatergic sPSCs at 4 weeks after induction, recorded in the presence of 100 μM PTX and blocked by addition of 5 μM NBQX and 50 μM APV. **(F)** Current trace illustrates the presence of GABAergic sPSCs at 4 weeks after induction, recorded in the presence of 5 μM NBQX and 50 μM APV and blocked by addition of 100 μM PTX.

The hES-iNs were able to generate action potentials (APs) upon current injection (Fig 5A, S2A Fig and S3A Fig). Maximum number of APs increased with time for N- and NF-derived hES-iNs (N: 4 weeks: 14±2; 8 weeks: 23±3, S2C Fig. NF: 4 weeks: 12±3; 8 weeks: 22±5 APs, Fig 5C), whereas it remained unaltered for NS-derived hES-iNs (NS: 5 weeks: 17±2; 8 weeks: 16±2, S3C Fig). This lack of change for NS-derived hES-iNs between 5 and 8 weeks suggests that these neurons, from an electrophysiological perspective, had reached their highest level of maturation already after 5 weeks of culturing. The AP characteristics remained the same or gained more mature features for N- and NF-derived hES-iNs, whereas AP characteristics except from AP threshold and AP rise time of NS-derived hES-iNs did not change with time in culture (Table 3). The APs could be blocked by the sodium channel blocker TTX (Fig 5A, S2A Fig and S3A Fig).

**Table 3:** Characteristics of APs in hES-iNs *in vitro*.

	N		NF		NS	
	4 weeks	8 weeks	4 weeks	8 weeks	5 weeks	8 weeks
n	6	6	7	6	7	7
AP threshold (mV)	-24 ± 2	-30 ± 2	-29 ± 3	-29 ± 3	-32 ± 2	-26 ± 1*
AP amplitude (mV)	51 ± 6	76 ± 3*	59 ± 5	69 ± 7	83 ± 4	73 ± 4
AP rise time (ms)	1.7 ± 0.3	0.8 ± 0.0*	1.5 ± 0.2	0.8 ± 0.1*	0.8 ± 0.1	1.1 ± 0.1*
½AP amp. width (ms)	1.7 ± 0.4	1.0 ± 0.1	1.7 ± 0.1	1.2 ± 0.2*	1.1 ± 0.1	1.1 ± 0.1
AHP (mV)	23 ± 3	20 ± 1	18 ± 3	24 ± 3	30 ± 2	33 ± 1

* indicates significant difference between 4–5 and 8 weeks.

Voltage-clamp recordings revealed the presence of both fast inward sodium current and a sustained outward potassium current (Fig 5D, S2D Fig and S3D Fig) in N-, NF- and NS-derived hES-iNs already from 4 weeks of culturing. After 8 weeks, there was a trend towards an enhanced fast inward sodium current in NF- and NS-derived hES-iNs, whereas the sustained outward potassium current remained stable (Fig 5D, S2D Fig and S3D Fig). The fast inward sodium current and the sustained outward potassium currents were inhibited by TTX and TEA, respectively (Fig 5D, S2D Fig and S3D Fig). Taken together, our electrophysiological findings indicate that N-, NF-, and NS-derived hES-iNs become functional neurons after 4 weeks of culturing. The NS-derived hES-iNs have reached their peak of maturation at 4 weeks, whereas N- and NF-derived hES-iNs continue to mature until 8 weeks in culture.

We next sought to determine whether the generated hES-iNs could form synaptic connections. In support, we observed spontaneous postsynaptic currents (PSCs) in many N-, NF-, and NS-derived hES-iNs, which had been co-cultured with mouse astrocytes for 8 weeks. The majority of recorded cells expressed glutamatergic PSCs, which were isolated in the presence of the GABA receptor blocker, PTX, and could be blocked by the NMDA and AMPA receptor antagonists APV and NBQX, respectively (Table 4, Fig 5, S2 Fig and S3 Fig). The frequency of glutamatergic PSCs was the same at 4–5 and 8 weeks of culturing for all hES-iNs (Table 4). A small subset of N-, NF-, and NS-derived hES-iNs exhibited GABAergic PSCs, which were isolated in the presence of APV and NBQX, and blocked by the presence of PTX (Fig 5, S2 Fig and S3 Fig). Our findings of both glutamatergic and GABAergic PSCs in the N-, NF-, and NS-derived hES-iNs provide evidence for their capacity to establish functional afferent synaptic inputs.

**Table 4:** Frequency of glutamatergic PSCs in hES-iNs *in vitro*.

	N		NF		NS	
	4 weeks	8 weeks	4 weeks	8 weeks	5 weeks	8 weeks
n	9	5	5	5	6	5
PTX	0.88 ± 0.27Hz	0.70 ± 0.41Hz	0.41 ± 0.23Hz	0.52 ± 0.18Hz	0.65 ± 0.17Hz	2.46 ± 1.81Hz
PTX+ APV+NBQX	0.01 ± 0.01Hz*	0.03 ± 0.01Hz*	0.01 ± 0.004Hz*	0.02 ± 0.01Hz*	0.00 ± 0.00Hz*	0.003 ± 0.003Hz*

* indicates significant decrease in PSC frequency upon APV and NBQX application.

#### Cortical neurons generated by three transcription factor combinations mature and become functional after grafting onto organotypic cultures of adult human cortex

**Fig 6:**
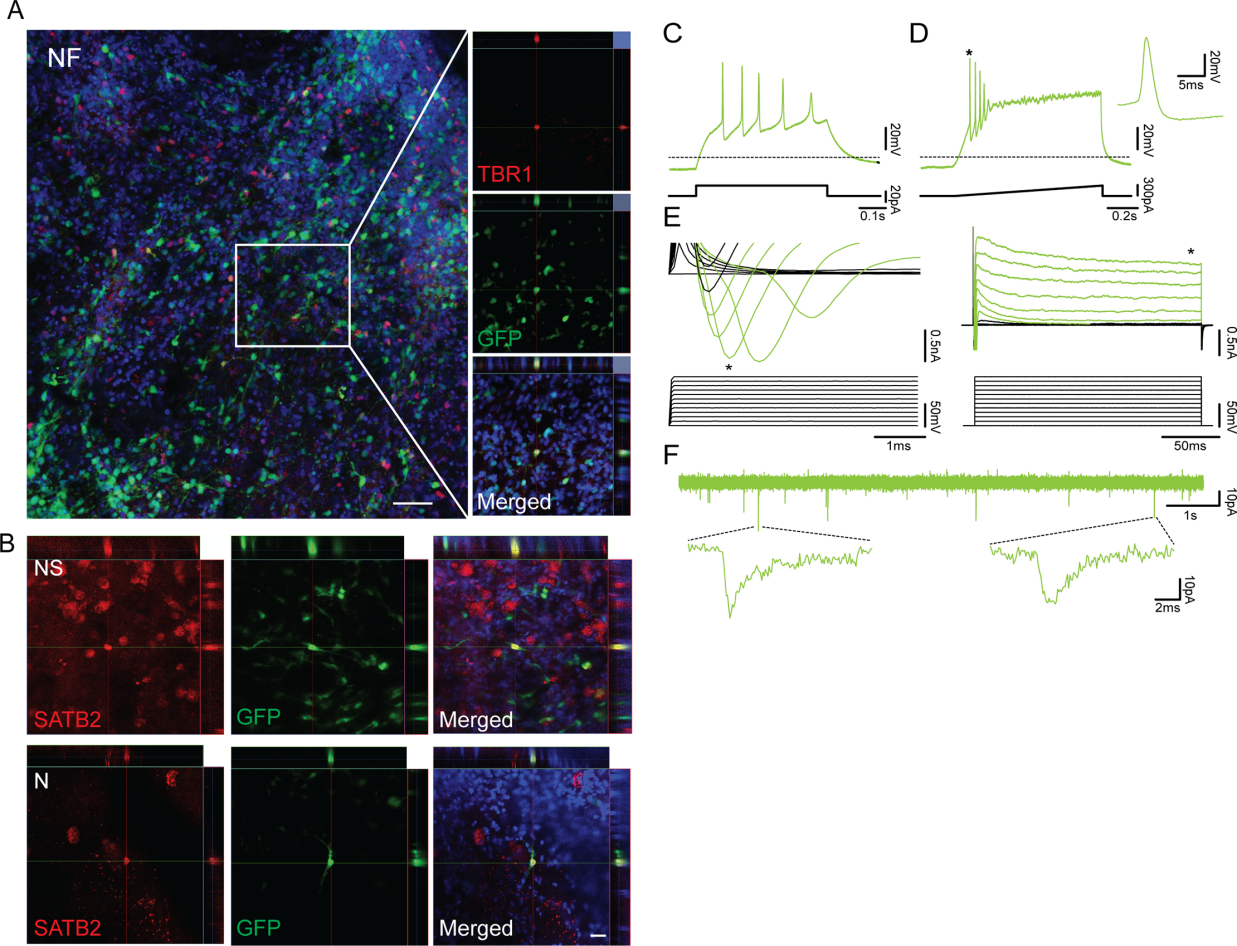
Human ES-iN cells derived by tree TF combinations integrate into adult human cortical tissue. **(A**) Confocal image showing an overview of NF-derived hES-iNs plated onto adult human cortical slices (*left*) and orthogonal projections of confocal images showing expression of the deep-layer cortical neuronal marker TBR1 (*right*). Scale bar: 50 μm. (**B**) Orthogonal projections of confocal images showing expression of the upper-layer cortical neuronal marker SATB2 in N- and NS-derived hES-iNs. Scale bar: 20 μm. **(C)** Whole-cell patch-clamp recordings from NF-derived hES-iNs at 5 weeks after transplantation onto hCtx organotypic slices (n = 7). Voltage traces illustrating the N-derived hES-iNs’ ability to generate APs during a 20 pA current step from a holding potential of -70mV. (**D**) Voltage trace illustrates APs generated during a current ramp from 0-300pA. * indicates expanded APs. **(E**) Expanded current traces illustrate the inward sodium current (*left*, denoted by *) and the outward sustained potassium current (*right*, denoted by *) activated during voltage steps ranging from -70 mV to +40 mV in 10 mV steps. **(F**) Current trace illustrates the presence of spontaneous downward deflecting currents.

To explore whether the generated hES-iNs are capable of integrating into human neural networks, we used transplantation onto organotypic slice cultures of adult human cortex. The cortical tissue was obtained from epileptic patients subjected to hippocampal resection surgery and handled as previously described [16]. Briefly, we first cultured the tissue slices for 4–6 days. From day 7, tetO-GFP labeled hES-iNs were plated on top of the cortical slices and kept in co-culture for 4 weeks. Immunohistochemistry revealed that many hES-iNs, derived by all three TF combinations, had survived and extended neurites throughout the adult human cortical tissue. The cells were covering not only the surface but could also be found deeper within the tissue as revealed by confocal microscopy. Some grafted NF-derived hES-iNs expressed the deep layer neuronal marker TBR1 (Fig 6A), whereas we found SATB2 positive cells in N- and NS-derived hES-iNs (Fig 6B).

We also performed whole-cell patch-clamp recordings on the grafted hES-iNs and found an averaged V_rest_ ranging from -47 mV to -49 mV (Table 5). The N-, NF-, and NS-derived hES-iNs were functional neurons, i.e., they had the ability to fire APs and express fast inward sodium current and outward sustained potassium currents, at 32–36 days after transplantation (Fig 6C–6F). The NS-derived hES-iNs seemed to fire more APs as compared to N- and NF-derived hES-iNs (Table 5). We observed spontaneous activity, most likely PSCs, in many of the N-, NF- and NS-derived hES-iNs (Fig 6C–6F and S4 Fig).

**Table 5:** Basic electrophysiological characteristics of hES-iNs transplanted onto adult human cortical organotypic slices.

	N	NF	NS
n	6	7	2
V_rest_	-49 ± 5 mV	-47 ± 5 mV	-47 ± 11 mV
R_input_	1582 ± 547 MΩ	2609 ± 571 MΩ	1653 ± 311 MΩ
C	3.0 ± 0.9 pF	1.4 ± 0.4 pF	1.7 ± 0.5 pF
Max # APs	3 ± 1 APs	2 ± 1 APs	11 ± 3 Aps

Taken together, these findings provide the first evidence that TF programmed cells can survive and integrate in the adult human brain.

## Discussion

Here we demonstrate that forced expression of three different combinations of the transcription factors NGN2, FEZF2 and SATB2 directs human ES cells to become pyramidal-shaped cells, morphologically resembling adult human cortical neurons, expressing cortical PN markers, and with mature electrophysiological properties. Our findings after grafting onto organotypic cultures suggest that these hES-iNs are also capable of integrating into adult human cortical neural circuitry. However, direct comparison between the different hES-iNs, derived with the three combinations of TFs, did not reveal any striking differences in either morphology, marker expression, molecular signature or electrical activity. Thus, our findings provided no evidence that the hES-iNs had acquired a distinct cortical layer phenotype depending on the TF combination used. Instead, our single-cell data showed that the hES-iNs expressed both upper and deep layer cortical neuronal markers.

Previous studies have indicated that the *in vitro* differentiation of human ES cells towards cortical neuronal fate follows a similar temporal order as observed in the intact developing cortex [6]. Neurons expressing deep layer markers occur first, followed by neurons having a phenotype of upper cortical layers. The method of TF programming used in the present study leads to accelerated differentiation of ES cells, at least in terms of their electrical activity [7, 8]. In accordance, we detected similar temporal cell maturation as reported earlier [7, 8] with newly derived hES-iNs from all three TF combinations exhibiting spontaneous activity as early as after 4 weeks in culture. Interestingly, we found that overexpression of an additional TF gave rise to even faster maturation of the NS-derived hES-iNs as compared to cells generated with N only. However, the strong intrinsic differentiation cues provided by overexpressing FEZF2 or SATB2 did not force the human ES cells expressing NGN2, which guides ES cells to an undefined excitatory neuronal phenotype [7], to become cortical neurons with specific layer identity. Only subtle differences in gene expression pattern and marker expression were observed between the hES-iNs generated by the three TF combinations. These findings were surprising since FEZF2 has been reported to be necessary and sufficient for cortical progenitors to generate deep-layer neurons and is specifically expressed in layer V neurons throughout life [10, 11]. FEZF2 has also been shown to reprogram layer IV neurons to layer V identity *in vivo* [12, 26]. In contrast, SATB2 is believed to be a regulator of upper layer cortical identity and has previously been demonstrated to be crucial for specifying callosal neuronal fate by repressing subcerebral identity upper layer neuronal cells [27, 28]. Also, accumulating evidence suggests that SATB2 is required for the development of subcerebral axons in a cell-context depending manner [9, 18].

Several potential explanations for why the TF combinations did not lead to generation of specific cortical layer phenotypes need to be considered: First, the timing of TF expression may have been incorrect. The development of cerebral cortex is tightly orchestrated in a temporal manner [29], with single progenitors sequentially generating different types of cortical neurons as shown by lineage tracing experiments [30–32]. Given that cell differentiation in the TF programming protocol follows a similar, only accelerated temporal order, it is conceivable that the human ES cells need not only the correct molecular cues but also the precise timing of their expression in order to generate specific subtypes of cortical neurons. Second, TF levels may have been suboptimal. During development, *Fezf2* is expressed at high levels by subcerebral projection neurons (SCPN) and at lower levels by corticothalamic projection neurons (CThPN) and subplate neurons. Also, TBR1 is mainly expressed postmitotically by CThPNs and subplate neurons but also at lower levels by CPNs in upper layers [33]. Conversely, SATB2 is expressed at a higher level in upper layer CPNs and lower level in CFuPNs during development [9]. Therefore, fine-tuning the levels of TFs in the human ES cells might be crucial for inducing a specific phenotype of the generated cortical neurons. Third, other signaling molecules and growth factors may also be necessary for subtype specification. Even if part of cortical neurogenesis is controlled cell-autonomously through TF expression, a number of extracellular cues are also involved. In particular, members of the classical morphogen families, i.e., retinoic acid, Wnts, bone morphogenic proteins/transforming growth factors, sonic hedgehog, and fibroblast growth factors are expressed throughout cortical development [34]. This raises the possibility that addition of a defined cocktail of morphogens would guide the human ES cells subjected to TF programming to acquire a specific neuronal subtype identity.

Our data from the single-cell analysis revealed that individual hES-iNs generated by all three TF combinations expressed canonical markers of both upper and deep cortical layers. This finding was unexpected given the well-established roles of NGN2, SATB2 and FEZF2 during embryonic development, driving progenitor fate to distinct cortical layer phenotypes *in vivo*. The complex upper/deep layer expression pattern of hES-iNs was not influenced by the used TF combination. With the accelerated functional maturation of hES-iNs, one may speculate whether the generated cells followed a normal temporal differentiation order with regard to their identity choice, or some steps of differentiation were not occurring at all, causing cellular fate ‘confusion’. The hES-iNs shared many features of their molecular signature with hFCtx^F^. However, neural progenitor markers were not expressed in hES-iNs, suggesting that they had advanced further into maturation compared to hFCtx^F^ cells. Importantly, we found that also single hFCtx^F^ cells expressed both upper and deep layer cortical markers. This finding argues against the hypothesis that the TF-induced programming of human ES cells and accelerated maturation had caused cellular fate “confusion.” In fact, recent data [35] indicate that mouse neural precursors are transcriptionally primed to make diverse cortical neuron subtypes with post-transcriptional mechanisms selecting when and where neuronal specification mRNAs are translated. For mouse cells, this transcriptional flexibility can persist even after neurogenesis is complete and neurons are committed to a single identity at the protein level [35]. In analogy with the findings of Zahr and co-workers, our single-cell data probably reflect that the hES-iNs generated here are still transcriptionally primed to make cortical neurons of diverse subtypes.

In summary, our results show that additional challenges, including testing of a broader range of TFs, have to be met in order to reach our long-term objective to generate pure populations of hES-iNs with specific cortical layer identity. We also conclude that analysis of multiple markers in single cells will be necessary to convincingly demonstrate the generation of *bona fide* subtypes of cortical neurons. It is likely that even if a correct combination of TFs is chosen, and they are expressed with precise timing and at optimum doses, also other molecules may have to be present in order to achieve this goal.

## Supporting information

S1 Fig Representative immunofluorescence images of N- and NS-derived hES-iNs and hFCtx^C^ cells. (**A**) Representative immunofluorescence images of N- and NS-derived hES-iNs expressing neuronal marker MAP2 at day 14 of induction. For N-derived hES-iNs see Fig 1C. Scale bar: 50 μm. (**B**) Representative immunofluorescence images of hFCtx^C^ cells expressing cortical PN markers SATB2 (*arrow*, *top panel*), BRAIN2 (*arrow head*, *lower panel*) and TBR1 (*arrow*, *lower panel*). Scale bar: 50 μm. (**C**) Representative immunofluorescence images of deep layer cortical marker TBR1 expressed in human adult cortical tissue. Scale bar: 20 μm. hACtx–adult human cortical tissue.
(TIF)

S2 Fig Whole-cell patch-clamp recordings performed from N-derived hES-iNs at 4 (n = 6) and 8 (n = 6) weeks after induction. (**A**) Representative voltage traces illustrating the N-derived hES-iNs’ ability to generate APs during a current step from a holding potential of -70mV at 4 (light blue) and 8 (dark blue) weeks after induction. APs were completely abolished in the presence of 1μM TTX (black traces). * indicates expanded APs. (**B**) Number of generated APs plotted against the current steps. (**C**) Bar diagram illustrating maximum number of APs generated during current steps (10–190 pA in 10 pA steps). * indicates significance (p < 0.05). (**D**) Expanded current traces illustrating the inward sodium current (*top*, denoted by *) and the outward sustained potassium current (*bottom*, denoted by *) activated during voltage steps ranging from -70 mV to +40 mV in 10 mV steps at 4 (light blue) and 8 (dark blue) weeks after induction. The sodium and the potassium current were blocked by the presence of 1 μM TTX (*top*, black) and 1μM TTX + 10 mM TEA (*bottom*, black), respectively. The plots illustrate the sodium current peak (*top*) and the outward potassium current (*bottom*) plotted against the voltage steps in the absence and presence of TTX and TTX+TEA, respectively. (**E**) Current trace illustrates the presence of glutamatergic sPSCs at 4 weeks after induction, recorded in the presence of 100 μM PTX and blocked by addition of 5 μM NBQX and 50 μM APV. (**F**) Current trace illustrates the presence of GABAergic sPSCs at 4 weeks after induction, recorded in the presence of 5 μM NBQX and 50 μM APV and blocked by addition of 100 μM PTX.
(TIF)

S3 Fig Whole-cell patch-clamp recordings performed from NS-derived hES-iNs at 5 (n = 7) and 8 (n = 7) weeks after induction. (**A**) Representative voltage traces illustrating the NS-derived hES-iNs’ ability to generate APs during a current step from a holding potential of -70mV at 5 (light red) and 8 (red) weeks after induction. Bottom trace illustrates APs generated during a current ramp from 0-300pA. APs were completely abolished in the presence of 1μM TTX (black traces). * indicates expanded APs. (**B**) Number of generated APs plotted against the current steps. (**C**) Maximum number of APs generated during current steps (10–390 pA in 10 pA steps). (**D**) Expanded current traces illustrate the inward sodium current (*top*, denoted by *) and the outward sustained potassium current (*bottom*, denoted by *) activated during voltage steps ranging from -70 mV to +40 mV in 10 mV steps at 4 (light red) and 8 (red) weeks after induction. The sodium and the potassium current were blocked by the presence of 1 μM TTX (*top*, black) and 1μM TTX + 10 mM TEA (*bottom*, black), respectively. The plots illustrate the sodium current peak (*top*) and the outward potassium current (*bottom*) plotted against the voltage steps in the absence and presence of TTX and TTX+TEA, respectively. (**E**) Current trace illustrates the presence of glutamatergic sPSCs at 5 weeks after induction, recorded in the presence of 100 μM PTX and blocked by addition of 5 μM NBQX and 50 μM APV. (**F**) Current trace shows the presence of GABAergic sPSCs at 8 weeks after induction, recorded in the presence of 5 μM NBQX and 50 μM APV and blocked by addition of 100 μM PTX.
(TIF)

S4 Fig Whole-cell patch-clamp recordings performed from N-and NS-derived hES-iNs at 5 weeks after transplantation onto adult human cortical organotypic slices (n = 6 and n = 2, respectively). (**A**) Voltage traces illustrating the N-derived hES-iNs’ ability to generate APs during a 50 pA current step from a holding potential of -70mV. (**B**) Voltage trace illustrates APs generated during a current ramp from 0-300pA. * indicates expanded APs. **C**) Expanded current traces illustrate the inward sodium current (*left*, denoted by *) and the outward sustained potassium current (*right*, denoted by *) activated during voltage steps ranging from -70 mV to +40 mV in 10 mV steps. **(D)** Current trace illustrates the presence of spontaneous downward deflecting currents. **(E)** Voltage traces illustrating the NS-derived hES-iNs’ ability to generate APs during a 50 pA current step from a holding potential of -70mV. **(F)** Voltage trace illustrates APs generated during a current ramp from 0-300pA. * indicates expanded APs. **(G)** Expanded current traces illustrate the inward sodium current (*left*, denoted by *) and the outward sustained potassium current (*right*, denoted by *) activated during voltage steps ranging from -70 mV to +40 mV in 10 mV steps. **(H)** Current trace illustrates the presence of spontaneous downward deflecting currents.
(TIF)
