# Supplementary figures and images for: Transcription factor programming of human ES cells generates functional neurons expressing both upper and deep layer cortical markers
Source: PLoS One. 2018 Oct 11;13(10):e0204688. doi: 10.1371/journal.pone.0204688 (PMC6181302; doi:10.1371/journal.pone.0204688)

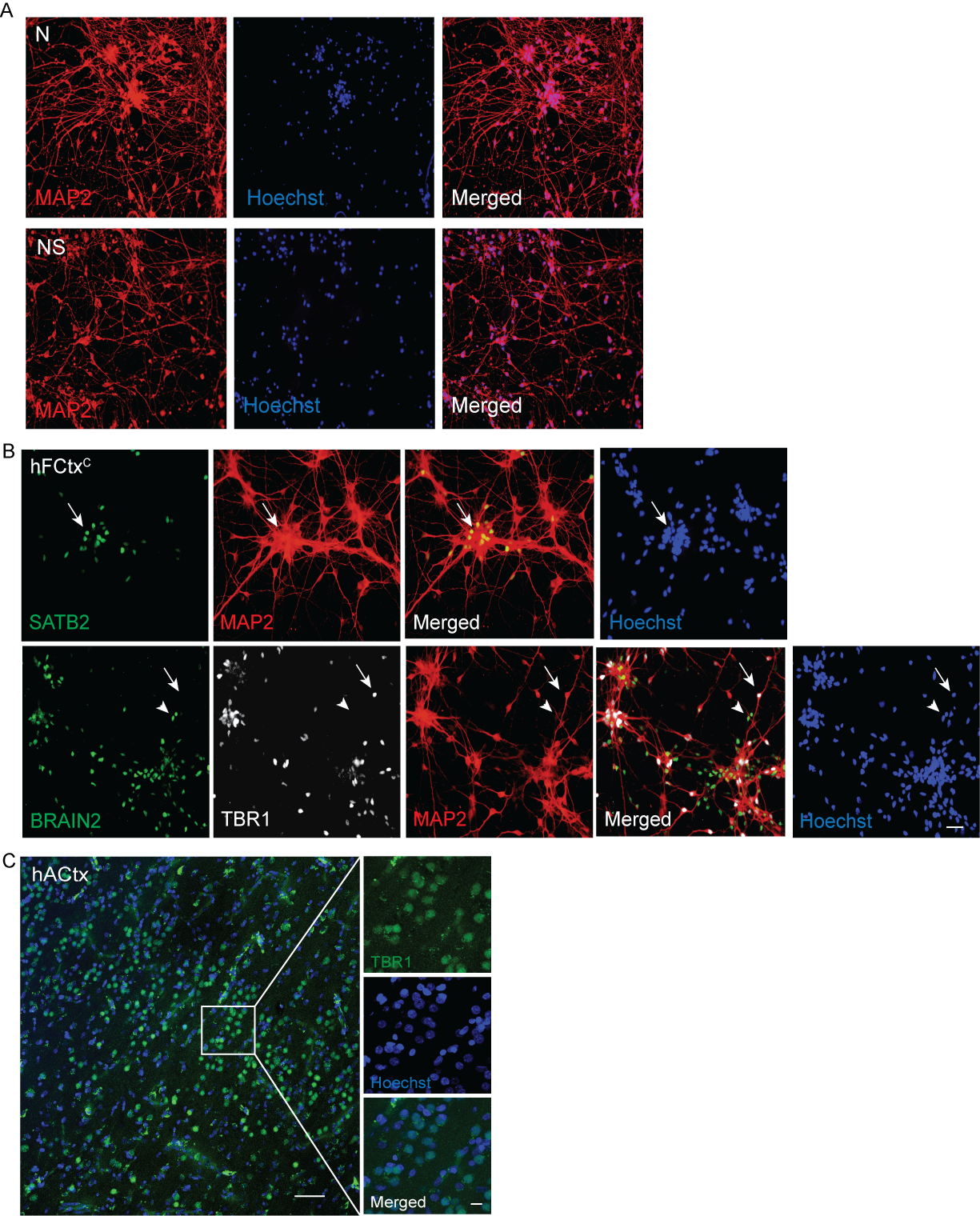

Supplement: S1 Fig — (A) Representative immunofluorescence images of N- and NS-derived hES-iNs expressing neuronal marker MAP2 at day 14 of induction. For N-derived hES-iNs see Fig 1C. Scale bar: 50 μm. (B) Representative immunofluorescence images of hFCtxC cells expressing cortical PN markers SATB2 (arrow, top panel), BRAIN2 (arrow head, lower panel) and TBR1 (arrow, lower panel). Scale bar: 50 μm. (C) Representative immunofluorescence images of deep layer cortical marker TBR1 expressed in human adult cortical tissue. Scale bar: 20 μm. hACtx–adult human cortical tissue. (TIF) [file pone.0204688.s001.tif]

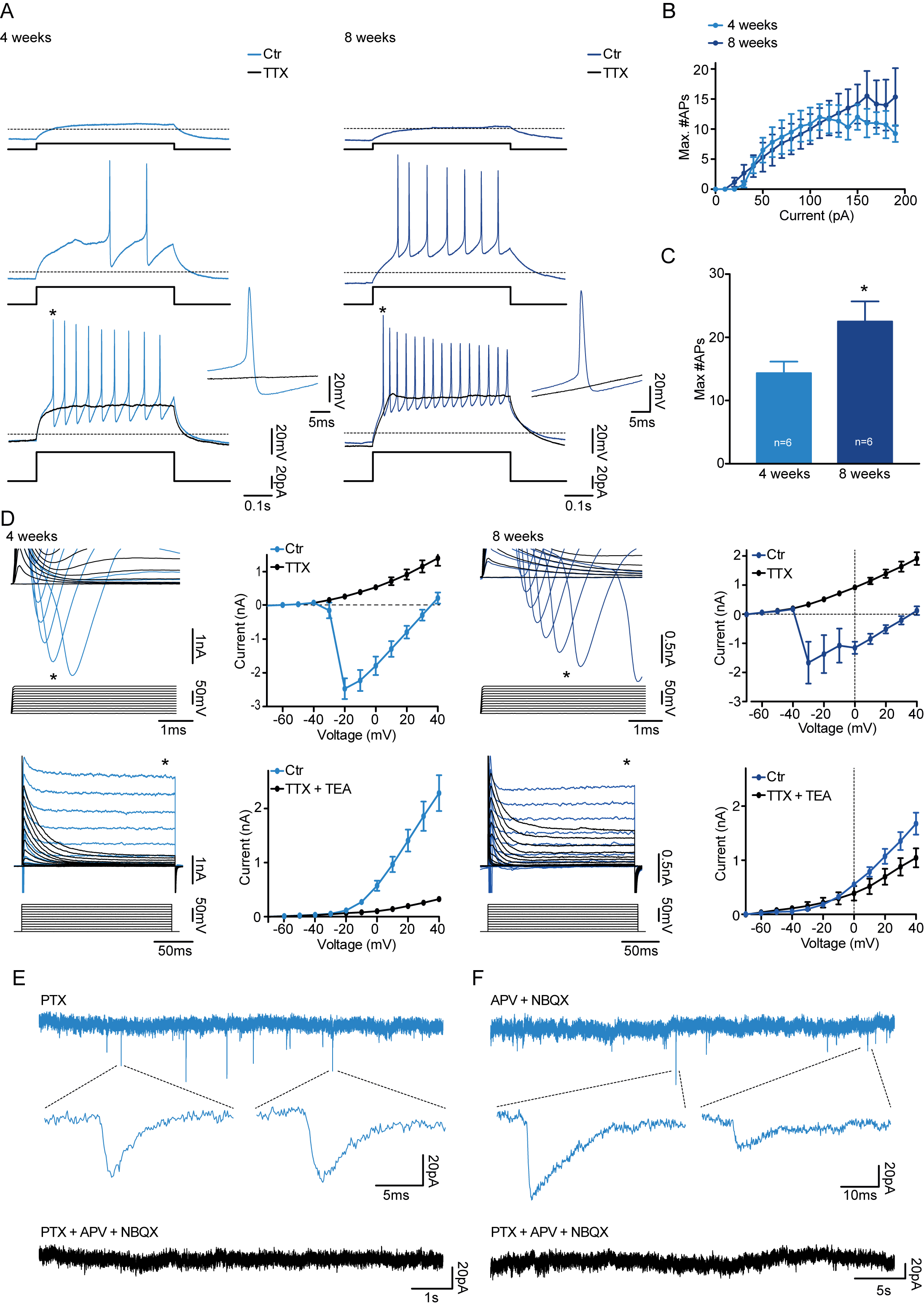

Supplement: S2 Fig — (A) Representative voltage traces illustrating the N-derived hES-iNs’ ability to generate APs during a current step from a holding potential of -70mV at 4 (light blue) and 8 (dark blue) weeks after induction. APs were completely abolished in the presence of 1μM TTX (black traces). * indicates expanded APs. (B) Number of generated APs plotted against the current steps. (C) Bar diagram illustrating maximum number of APs generated during current steps (10–190 pA in 10 pA steps). * indicates significance (p < 0.05). (D) Expanded current traces illustrating the inward sodium current (top, denoted by *) and the outward sustained potassium current (bottom, denoted by *) activated during voltage steps ranging from -70 mV to +40 mV in 10 mV steps at 4 (light blue) and 8 (dark blue) weeks after induction. The sodium and the potassium current were blocked by the presence of 1 μM TTX (top, black) and 1μM TTX + 10 mM TEA (bottom, black), respectively. The plots illustrate the sodium current peak (top) and the outward potassium current (bottom) plotted against the voltage steps in the absence and presence of TTX and TTX+TEA, respectively. (E) Current trace illustrates the presence of glutamatergic sPSCs at 4 weeks after induction, recorded in the presence of 100 μM PTX and blocked by addition of 5 μM NBQX and 50 μM APV. (F) Current trace illustrates the presence of GABAergic sPSCs at 4 weeks after induction, recorded in the presence of 5 μM NBQX and 50 μM APV and blocked by addition of 100 μM PTX. (TIF) [file pone.0204688.s002.tif]

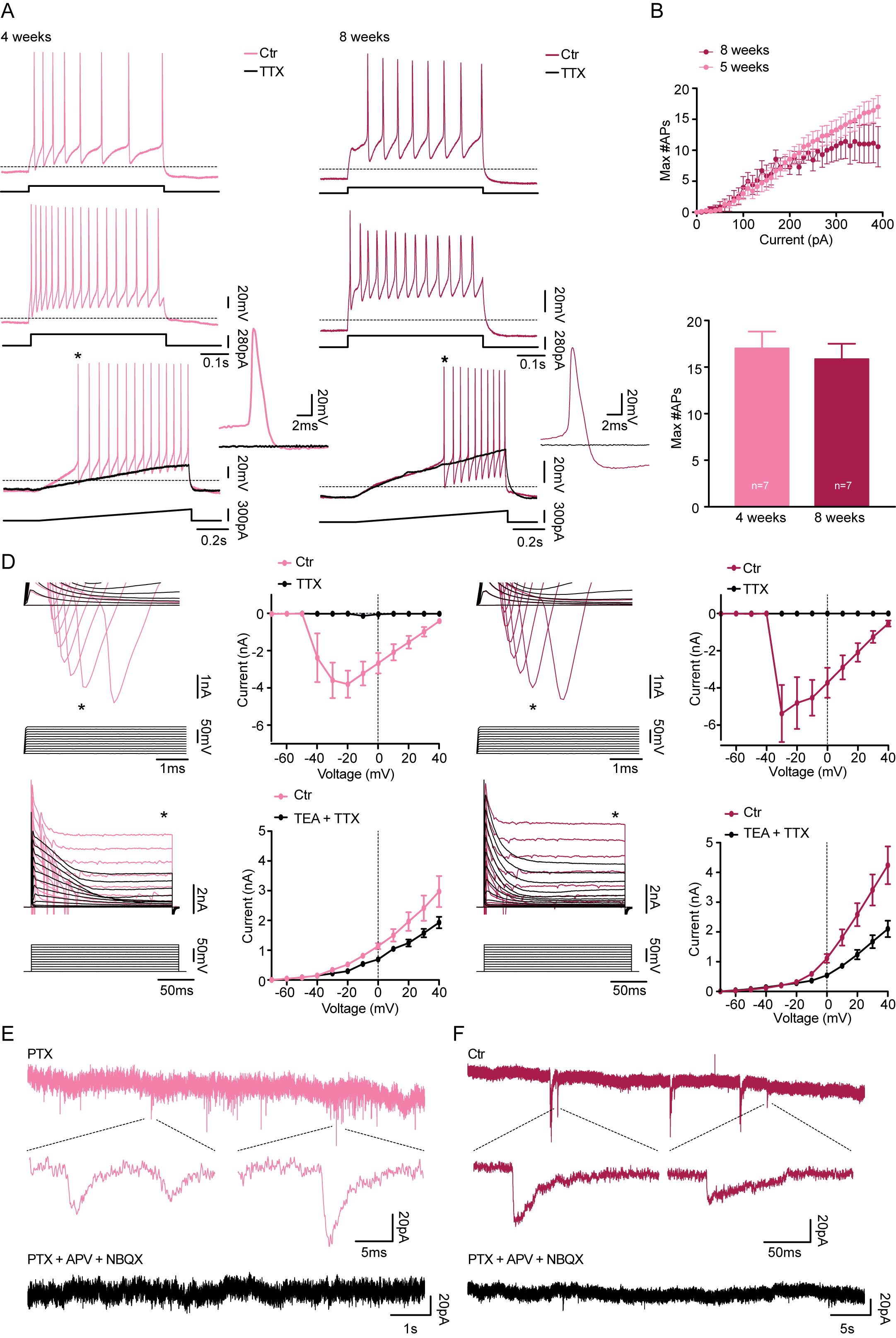

Supplement: S3 Fig — (A) Representative voltage traces illustrating the NS-derived hES-iNs’ ability to generate APs during a current step from a holding potential of -70mV at 5 (light red) and 8 (red) weeks after induction. Bottom trace illustrates APs generated during a current ramp from 0-300pA. APs were completely abolished in the presence of 1μM TTX (black traces). * indicates expanded APs. (B) Number of generated APs plotted against the current steps. (C) Maximum number of APs generated during current steps (10–390 pA in 10 pA steps). (D) Expanded current traces illustrate the inward sodium current (top, denoted by *) and the outward sustained potassium current (bottom, denoted by *) activated during voltage steps ranging from -70 mV to +40 mV in 10 mV steps at 4 (light red) and 8 (red) weeks after induction. The sodium and the potassium current were blocked by the presence of 1 μM TTX (top, black) and 1μM TTX + 10 mM TEA (bottom, black), respectively. The plots illustrate the sodium current peak (top) and the outward potassium current (bottom) plotted against the voltage steps in the absence and presence of TTX and TTX+TEA, respectively. (E) Current trace illustrates the presence of glutamatergic sPSCs at 5 weeks after induction, recorded in the presence of 100 μM PTX and blocked by addition of 5 μM NBQX and 50 μM APV. (F) Current trace shows the presence of GABAergic sPSCs at 8 weeks after induction, recorded in the presence of 5 μM NBQX and 50 μM APV and blocked by addition of 100 μM PTX. (TIF) [file pone.0204688.s003.tif]

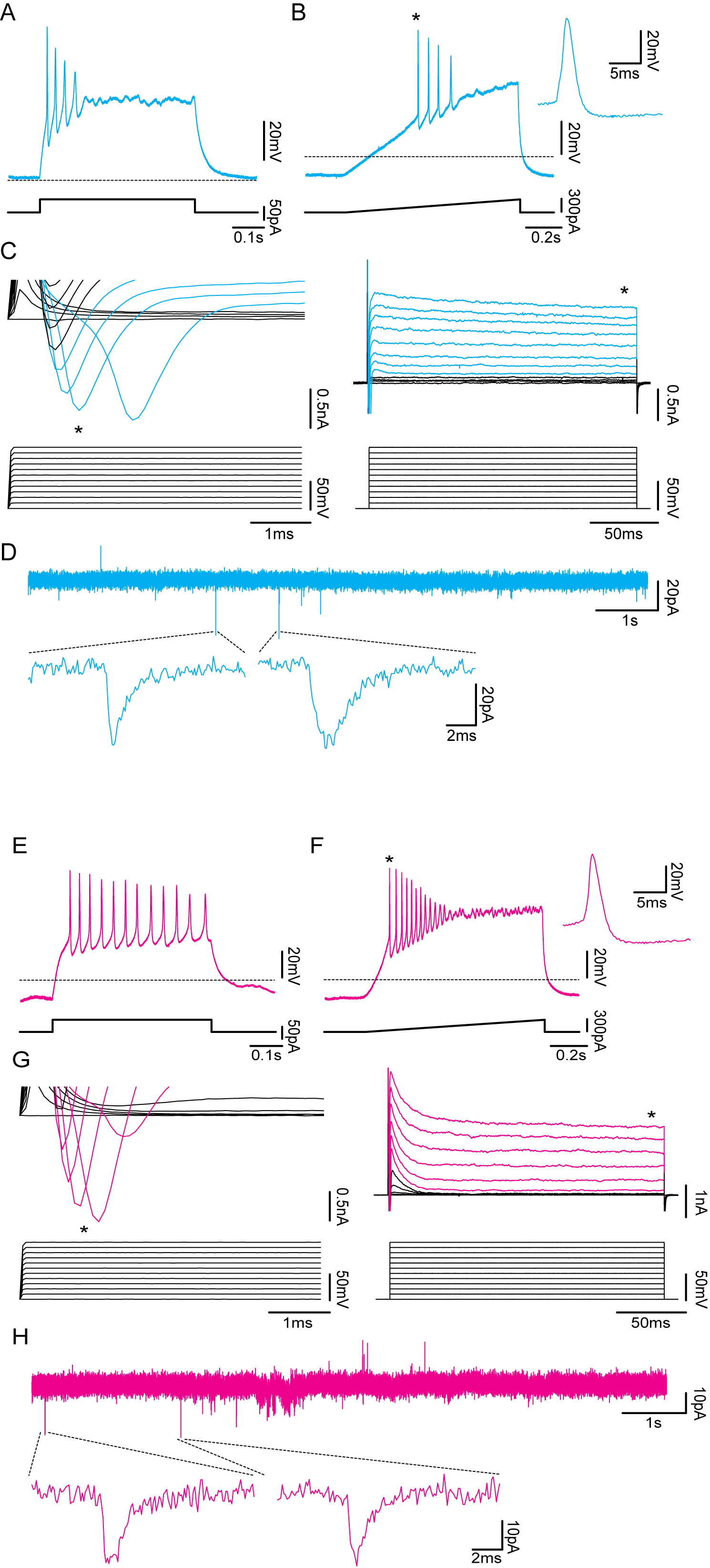

Supplement: S4 Fig — (A) Voltage traces illustrating the N-derived hES-iNs’ ability to generate APs during a 50 pA current step from a holding potential of -70mV. (B) Voltage trace illustrates APs generated during a current ramp from 0-300pA. * indicates expanded APs. C) Expanded current traces illustrate the inward sodium current (left, denoted by *) and the outward sustained potassium current (right, denoted by *) activated during voltage steps ranging from -70 mV to +40 mV in 10 mV steps. (D) Current trace illustrates the presence of spontaneous downward deflecting currents. (E) Voltage traces illustrating the NS-derived hES-iNs’ ability to generate APs during a 50 pA current step from a holding potential of -70mV. (F) Voltage trace illustrates APs generated during a current ramp from 0-300pA. * indicates expanded APs. (G) Expanded current traces illustrate the inward sodium current (left, denoted by *) and the outward sustained potassium current (right, denoted by *) activated during voltage steps ranging from -70 mV to +40 mV in 10 mV steps. (H) Current trace illustrates the presence of spontaneous downward deflecting currents. (TIF) [file pone.0204688.s004.tif]
